# Colonic mucosal and cytobrush sample cytokine mRNA expression in canine inflammatory bowel disease and their correlation with disease activity, endoscopic and histopathologic score

**DOI:** 10.1371/journal.pone.0245713

**Published:** 2021-01-20

**Authors:** Alexandros O. Konstantinidis, Katerina K. Adamama-Moraitou, Dimitra Pardali, Chrysostomos I. Dovas, Georgia D. Brellou, Theologos Papadopoulos, Albert E. Jergens, Karin Allenspach, Timoleon S. Rallis

**Affiliations:** 1 Companion Animal Clinic (Medicine Unit), School of Veterinary Medicine, Faculty of Health Sciences, Aristotle University of Thessaloniki, Thessaloniki, Greece; 2 Diagnostic Laboratory, School of Veterinary Medicine, Faculty of Health Sciences, Aristotle University of Thessaloniki, Thessaloniki, Greece; 3 Laboratory of Pathology, School of Veterinary Medicine, Faculty of Health Sciences, Aristotle University of Thessaloniki, Thessaloniki, Greece; 4 Department of Clinical Sciences, Iowa State University College of Veterinary Medicine, Ames, IA, United States of America; University of Illinois at Chicago, UNITED STATES

## Abstract

Canine inflammatory bowel disease (IBD) is a group of chronic gastrointestinal disorders, the pathogenesis of which remains elusive, but it possibly involves the interaction of the intestinal immune system with luminal microbiota and food-derived antigens. Mucosal cytokines profiles in canine IBD have been investigated mainly in small intestinal disease, while data on cytokine profiles in large intestinal IBD are limited. The objective of this study was to measure colonic mucosal and cytobrush sample messenger (m)RNA expression of interleukin (IL)-1β, IL-2, IL-12p40, IL-23p19, tumor necrosis factor-alpha (TNF-α) and chemokine C‐C motif ligand (CCL28) in dogs with IBD and healthy controls using quantitative real-time polymerase chain reaction (PCR), and assess their correlation with clinical disease activity, endoscopic and histopathologic score. Dogs with IBD had a significantly increased mRNA expression of IL-1β, IL-23p19 and CCL28 in the colonic mucosa, compared to healthy controls. None of the selected cytokines had significantly different mRNA expression in the colonic cytobrush samples between the two groups or between the colonic mucosa and cytobrush samples of dogs with IBD. Finally, there was a statistically significant correlation of clinical disease activity with endoscopic activity score and fibrosis and atrophy of the colonic mucosa in dogs with large intestinal IBD. IL-1β, IL-23p19 and CCL28 could play a role in the pathogenesis of canine large intestinal IBD. Colonic cytokine expression does not correlate with clinical disease activity and/or endoscopic score. However, clinical signs reflect the severity of endoscopic lesions.

## Introduction

Inflammatory bowel disease (IBD) in dogs is a group of chronic gastrointestinal (GI) disorders affecting the small and/or large intestine, with persistent or relapsing clinical signs and histopathologic evidence of inflammation [1, 2]. The etiopathogenesis of IBD is still elusive, but it possibly involves the interaction of the intestinal immune system with luminal microbiota and food-derived antigens [3].

Cytokines play a key role in the modulation of the mucosal immune system. Different studies have failed to identify distinct cytokine profiles in canine IBD (mixed Th1 and Th2 derived cytokine response) [4–10]. The vast majority of these studies investigated cytokine signature in the small intestine of dogs with IBD; thus, data on cytokine expression in canine large intestinal IBD are limited, with only one study using a quantitative method [6]. More specifically, two studies investigated colonic mucosal cytokine gene expression using a semi-quantitative polymerase chain reaction (PCR) method [4, 7]. Ridyard et al. found increased interleukin (IL)-2 and tumor necrosis factor-alpha (TNF-α) messenger (m)RNA expression in the colonic mucosa of dogs with IBD compared to healthy controls (Th1 immune response) [7]. To the contrary, Jergens et al. reported that dogs with large intestinal IBD failed to demonstrate a Th1 or Th2 polarization [4]. Finally, Tamura et al. in a recent study using a quantitative real- time PCR method, did not find a predominant cytokine profile in the colonic mucosa of a small group of dogs with large intestinal IBD [6].

The GI tract is covered by an epithelial mucus layer, which is the product of secretory cells forming the luminal adjacent layer of the mucosa. The large intestine is protected by a double mucus layer, composed of water, glycoproteins, lipids, proteins, salts, ions, cytokines, cellular macromolecules (e.g., secretory immunoglobulin A), and cellular debris [11–13]. Inflammation of the colonic mucosa leads to increased mucus production [14]. Canine IBD is clinically characterized by dysregulated mucus production and mucosal damage [1, 14–17]. Mucosal erythema, friability, enhanced granularity, ulceration and erosions are often seen during colonoscopy. Based on clinical and endoscopic observations, combined with the potential advantages of the colonic cytobrush samples (more representative sampling, as they cover a larger mucosa surface, and less invasive compared to mucosal biopsies), we hypothesized that these mucosal lesions may also reflect altered cytokines mRNA expression in colonic cytobrush samples of dogs with IBD.

The aim of the present study was to investigate the colonic mucosal and cytobrush sample mRNA expression of IL-1β, IL-2, IL-12p40, IL-23p19, TNF-α and C‐C motif chemokine ligand (CCL28), using real-time RT-PCR, as well as the correlations of mRNA expression in the colonic mucosa with the cytobrush samples of dogs with large intestinal IBD. In addition, correlations between clinical disease activity, endoscopic and histopathologic score as well as their correlation with cytokine mRNA expression in the colonic mucosa were also evaluated. The selection of the cytokines in the current investigation was based on their potential role in large intestinal IBD pathogenesis and disease severity. More specifically, IL-1β is a proinflammatory cytokine that plays an important role in the pathogenesis of intestinal inflammation in human IBD with elevations of IL-1β levels being associated with increased IBD severity [18, 19]. Previous studies in canine IBD have shown increased IL-1β mRNA expression and protein levels, as well as increased IL-1β/ IL-1Ra protein ratio in the duodenal and/or colonic mucosa of dogs with IBD compared to healthy controls [20–24]. IL-2 and TNF-α expression has been previously studied in canine IBD using different methods with conflicting results [4–7, 9, 25]. In humans, IL-23 is associated with IBD pathogenesis, as it is upregulated during the disease process [26]. In addition, polymorphisms of the genes encoding IL-23 receptor and the IL-12p40 subunit are associated with increased susceptibility to IBD [27]. Furthermore, IL-23p19 expression has been associated with disease severity in human IBD [28]. Data regarding IL-23p19 expression in canine IBD are limited [6]. Finally, chemokines likely play a role in canine IBD pathogenesis, although data regarding their expression in canine IBD are limited and involve only small intestinal mucosal disease [29]. CCL28 is expressed mainly in the epithelial cells of the colon and could play an important role in canine large intestinal IBD.

## Materials and methods

### Dogs and study design

#### Dogs with IBD

26 dogs presented or referred to Companion Animal Clinic, School of Veterinary Medicine, Aristotle University of Thessaloniki for investigating large intestinal disease and were diagnosed with IBD. The dogs with large intestinal IBD described here were also used in another study [30]. Selection criteria, as previously described [30], included (1) a history of chronic (>3 weeks) large intestinal disease (large intestinal diarrhea with mucus, hematochezia, tenesmus, and/or increased frequency of defecation) without any identifiable underlying cause, (2) histopathologic evidence of intestinal inflammatory cellular infiltration and (3) minor or no response to a 3-week dietary trial (novel or hydrolyzed protein diets) to rule out adverse food reactions. Exclusion criteria were (1) the presence of a concurrent disease and (2) administration of any medication for a 2-week period prior to presentation or referral. The experimental protocol was reviewed and approved by the ethics committee of the School of Veterinary Medicine, Aristotle University of Thessaloniki (58/ 29-9-2015). Owners provided informed written consent for enrollment of their dogs to the study. The diagnostic evaluation performed in all dogs in order to exclude other known causes of large intestinal diarrhea included physical examination, complete blood count, routine serum biochemistry, serum folate and cobalamin concentrations, serum cPLI concentration (SNAP® cPL Test, IDEXX Laboratories, Westbrook, ME, USA), detection of Anti-Leishmania infantum antibodies in serum (Leishmania SNAP® Test, IDEXX Laboratories, Westbrook, ME, USA), detection of Anti-Anaplasma phagocytophilum/Anaplasma platys, Anti-Borrelia burgdorferi, and Anti-Ehrlichia canis antibodies and the Dirofilaria immitis antigen in serum (4DX SNAP® Test, IDEXX Laboratories, Westbrook, ME, USA), ACTH stimulation test, urinalysis, abdominal radiographic and ultrasonographic examination, parasitological and cytological examination of feces and detection of Giardia-specific cyst wall antigen in feces (SNAP® Giardia Test, IDEXX, Laboratories, Westbrook, ME, USA). Clinical disease activity was recorded for all dogs using the CCECAI scoring system [2], which is based on nine variables that are routinely evaluated in affected dogs: attitude and activity, appetite, vomiting, stool consistency, stool frequency, weight loss, ascites, pruritus and serum albumin concentration. The total CCECAI score was considered clinically insignificant (score 0–3), mild (score 4–5), moderate (score 6–8), severe (score 9–11), or very severe (score ≥ 12) [2]. For statistical purposes, dogs with clinically insignificant, mild and moderate CCECAI scores (score 0–8) were further categorized as the low CCECAI group and dogs with severe and very severe CCECAI scores (score ≥9) as the high CCECAI group. All dogs with IBD were enrolled in the study while they were symptomatic (active disease).

#### Healthy dogs (controls)

As healthy control dogs, 9 adult dogs with no GI signs for at least 6 months prior to diagnostic evaluation were used. Control dogs were judged to be healthy on the basis of normal results of physical examination, complete blood count, routine serum biochemistry, urinalysis, and parasitological and cytological examination of feces, as well as determination of Anti-Leishmania infantum, Anti-Anaplasma phagocytophilum/Anaplasma platys, Anti-Borrelia burgdorferi, and Anti-Ehrlichia canis antibodies and Dirofilaria immitis antigen in serum. Colonoscopy accompanied by colonic mucosal biopsy specimens and cytobrush samples collection for cytokines mRNA expression analysis. All dog owners provided informed written consent permitting enrollment of their dog into the study.

### Sample collection

Dogs were prepared for colonoscopy by withholding food for 48 hours and administering an osmotic laxative (Klean Prep®, Norgine B.V., Amsterdam, Netherlands). Colonoscopies were performed using a CF-140L flexible video endoscope (Olympus®, Hamburg, Germany). Multiple mucosal biopsies of the colon, from areas with macroscopic lesions, were collected using single-use multiple sample biopsy forceps (Multibite™; Boston Scientific, Marlborough, MA, USA) (9 for histopathologic examination and 4 for cytokine mRNA expression analysis). Two cytobrush samples from each dog were collected using endoscopic cytobrushes (Cellebrity™, Boston Scientific, Marlborough, USA). Samples were obtained by immersing the brush into the mucus at sites with macroscopic lesions and excessive mucus deposition. Mucosal brushings were performed using long or broad strokes, applying gentle pressure against the colonic mucosa, while taking care to avoid further injury of the mucosa and bleeding. Cytobrushes captured abraded material (mucus and colonic mucosal epithelium in dogs with IBD). After sampling, the cytobrush was retracted into its sterile sheath prior to withdrawal. In case of mucosal injury samples were discarded and the procedure was repeated. Endoscopic findings were graded according to the guidelines proposed by Slovak et al. (quantitative assessment) [31]. Three endoscopic parameters (friability, granularity and erosions) were scored (0, absent; 1, mild-to-moderate abnormal appearance; 2, moderate-to-severe abnormal appearance) in each case, with a maximum total score of 6 [31].

Biopsy samples were placed in 10% neutral buffered formalin for 48 hours, processed for histopathology examination and stained with H&E. Histopathological diagnoses of IBD and grading were performed according to the World Small Animal Veterinary Association (WSAVA) GI histopathologic guidelines [15], which characterizes the nature and number of infiltrative cells in the mucosa (lymphocytes and plasma cells, neutrophils, eosinophils and macrophages) as well as mucosalarchitectural changes (surface epithelial injury, crypt hyperplasia, crypt dilatation and distortion, fibrosis and atrophy). The above parameters were graded on a scale 0–3 in each case (0, absent; 1, mild; 2, moderate and 3 marked). Histopathologic examination was performed by a single pathologist (GDB) that was blinded to the clinical activity and endoscopic score of each dog.

### RNA isolation, complementary DNA synthesis, and quantification of cytokine mRNA transcription by real time RT-PCR

Four endoscopic biopsy specimens (20 mm^3^) and two cytobrush samples (0.1 ml) obtained from each dog were immediately processed using the TRIzol^TM^ Plus RNA purification kit (Thermo Fisher Scientific, Waltham, MA, USA) and genomic DNA was removed from the samples with RNase-Free DNase set kit (Qiagen Inc., Hilden, Germany). The reverse transcription (RT) reaction mixture (20 μl) contained 1μl first-strand buffer, 0.01 M dithiothreitol (DTT), 0.5 mM of each deoxynucleoside triphosphate (dNTP), 1.4 μM random hexamers, 50 U M-MLV reverse transcriptase, 13 U RNase inhibitor, 12 μl of RNA extract and RNase-free water (all reagents were obtained from Thermo Fisher Scientific, Waltham, CA, USA). Specific primers for quantitative PCR (qPCR) were designed based on the canine GenBank sequences. Specific primers targeted IL-1β, IL-2, IL-23p19, TNF-α, CCL28 and IL-12p40. Succinate dehydrogenase complex, subunit A (*SDHA*) reference gene was selected as appropriate housekeeping gene according to previous studies [6, 32]. The sequences of primer pairs are shown in Table 1. Primer pair specificities were confirmed by sequencing each respective amplicon using an ABI PRISM 3100 Genetic Analyzer (Applied Biosystems, Foster city, CA, USA). The qPCR reaction (20 μl) was comprised of 1× PCR buffer (Invitrogen, Carlsbad, CA, USA), 0.2 μM of each primer, 0.2 mM of each dNTP, 3 mM MgCl_2_, 3 U of Platinum™ Taq DNA Polymerase (Invitrogen, Carlsbad, CA, USA), 1× EvaGreen™ dye (Biotium, Hayward, CA, USA) and 2 μl of cDNA. The following thermal cycling conditions were applied: 94°C for 2 min, followed by 47 cycles in 2 steps: a) 95°C for 15 s and b) 60°C for 50 s. Fluorescence was recorded at the end of each cycle. After the completion of cycles, a melting curve was generated, by heating from 75°C to 90°C, in increments of 0.2°C/6 sec. Reactions were carried out on a CFX96 Touch™ Real-Time PCR Detection System (Bio-Rad Laboratories, Hercules, USA). Reaction efficiencies were determined for each primer set, using 10-fold serial dilutions (2*107–2*10^1^ copies/reaction) of plasmids ligated with each amplicon (Table 1). Melting curve analysis did not show nonspecific amplicons in any of the reactions. All samples were examined in triplicate, and each PCR reaction included a non-template control.

**Table 1:** Sequences of oligonucleotide primers used for quantitative real-time RT-PCR.

Primer set	Primer sequence (5’-3’)	Product size (bp)	Reaction efficiency (%)	Amplicon melting temperature (°C)
**IL-1β**	IL-1bF: 5’-GCAAGTCTCCCACCAGCTCTGTA-3’ IL-1bR: 5’-GGCAGGGCTTCTTCAGCTTCTCCA-3’	86	99	82
**IL-2**	IL-2F: 5’-ACCTGGAGGAAGTGCTAGGTTT-3’ IL-2R: 5’-TCTGTAATGGTTGCTGTCTCGTCA-3’	158	90	80.5
**IL-12p40**	IL-12p40F: 5’-TGCATCCTCAGCAGTTGGTCATC-3’ IL-12p40R: 5’- TCAGGGTGCCAGTCCAACTCTAC-3’	118	93	83.6
**IL-23p19**	IL-23p19F: 5’-GCTCTCACAGAAGCTCTGCACGC-3’ IL-23p19R: 5’-TCACTTGTAGTCTCACCATCTCCCTC-3’	78	99	84.7
**TNF-α**	TNF-aF: 5’-CCCTCTTGCCCAGACAGTCAAATCATCTT-3’ TNF-aR: 5’-GAGGTACAACCCATCTGACGGCACTA-3’	187	90	88.4
**CCL28**	CCL28F: 5’-CAGACAGGACTCACTCTCGCTCTC-3’ CCL28R: 5’-TGATGTGAAACCTCAGTGCAACAGC-3’	110	90	84.6
**SDHA**	SDHA F: 5’- ACTTTGCCTTGGATCTCTTGATGGA -3’ SDHAR: 5’-ACTCCTTGGAGGCCATGTAGAC-3’	102	99	81.2

Data was exported to Q-gene [33], adjusted for amplification efficiency and normalized to *SDHA*. Relative expression of a gene in each sample was calculated as arbitrary units (AU) by dividing its mean normalized expression by the average level of respective measurements in control samples [34].

### Statistical analysis

Statistical analysis was performed with IBM SPSS 19 (USA, Chicago, Illinois). Cytokine mRNA relative expression values were not normally distributed, so they were logarithmically transformed and assessed for normal distribution. Fisher’s exact test was used to compare sex between dogs with IBD and control dogs, as well as between low and high CCECAI group. The Mann-Whitney test was used to compare age and weight between dogs with IBD and control dogs, as well as between low and high CCECAI score groups. Comparison of cytokines relative mRNA expression levels between healthy controls and dogs with large intestinal IBD as well as between the low and high CCECAI score dogs with large intestinal IBD were made using t-tests (IL-23p19, TNF-α and CCL28) or Mann-Whitney tests (IL-1β, IL-2 and IL-12p40). Spearman correlation coefficient test was used to assess the relationship between cytokines relative mRNA expression in the colonic mucosa and cytobrush samples, between cytokines relative mRNA expression in the colonic mucosa with the CCECAI score the colonoscopy score and histopathologic parameters score and among CCECAI score the colonoscopy score and histopathologic parameters score. P values of < 0.05 were considered significant.

## Results

### Characteristics of dogs with IBD and healthy controls

Population demographic data, CCECAI and endoscopy score of dogs with active large bowel IBD (n = 26) included in this prospective study are presented in S1 Table. The median age of dogs with IBD at diagnosis was 7 years (range = 1–15 years) and a significant number of these were mongrels (7/26). Body weight ranged from 1.72 to 36.5 kg (median = 8 kg). Population demographic data of control dogs are shown in S2 Table. The median age of control dogs was 3.2 years (range = 2–7 years), most of them were mongrels (8/9) and their body weight ranged from 26 to 35 kg (median = 28 kg). There was no statistically significant difference in sex between the dogs with IBD and control dogs, as well as between the low and high severity CCECAI group. Statistically significant differences were found for age (*p* = 0.015) and weight (*p* < 0.0005) between dogs with IBD and control dogs. Finally, there were no statistically significant differences in age and weight between dogs with low and high severity CCECAI score.

**Fig 1:**
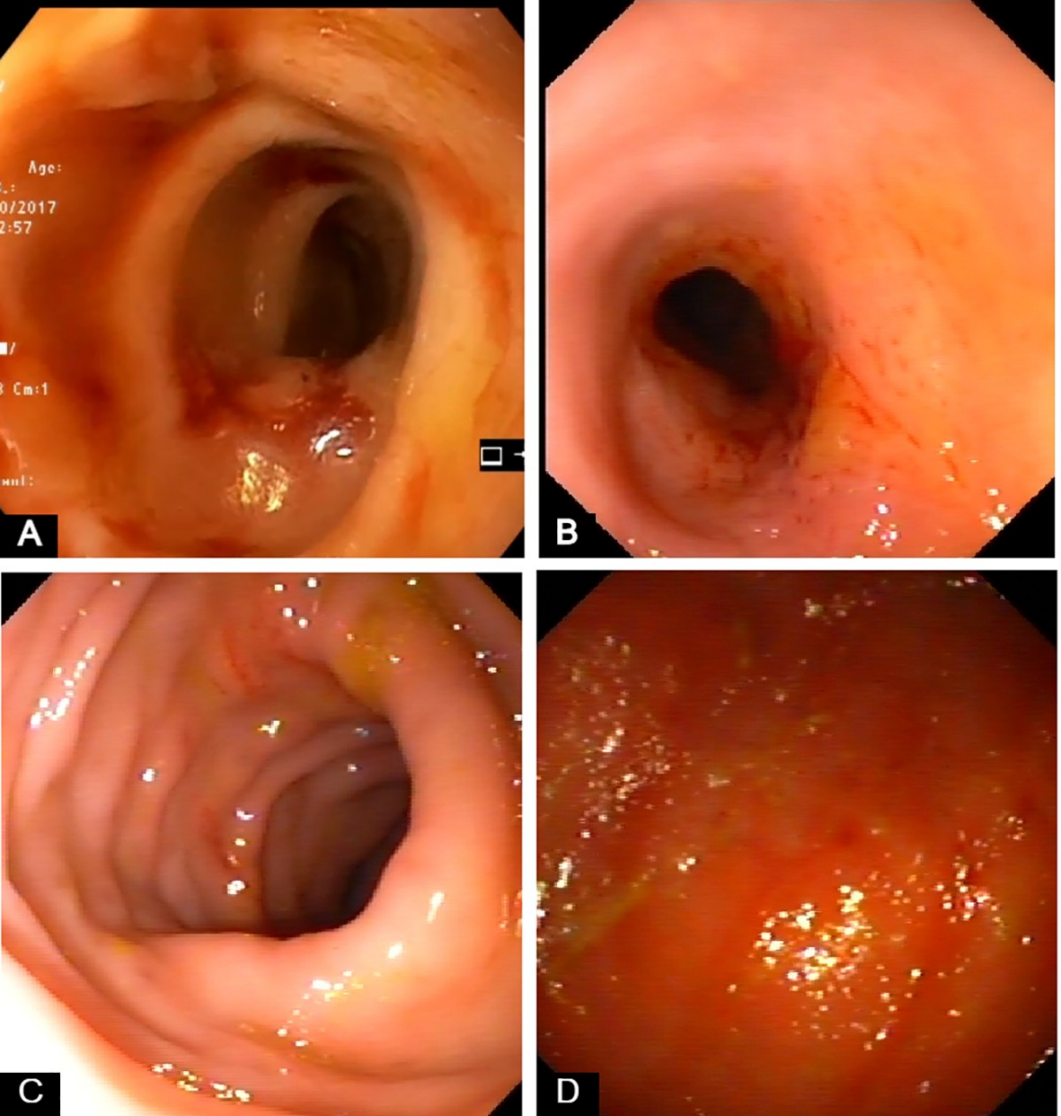
Representative endoscopic images from dogs with large intestinal inflammatory bowel disease (IBD) included in the study. (A) Marked friability, increased granularity and erosions. (B) Increased friability, granularity and erosions. Multiple superficial ulcers. (C) Increased erosions (D) Increased granularity and friability. Hyperemic and ulcerated mucosa.

**Fig 2:**
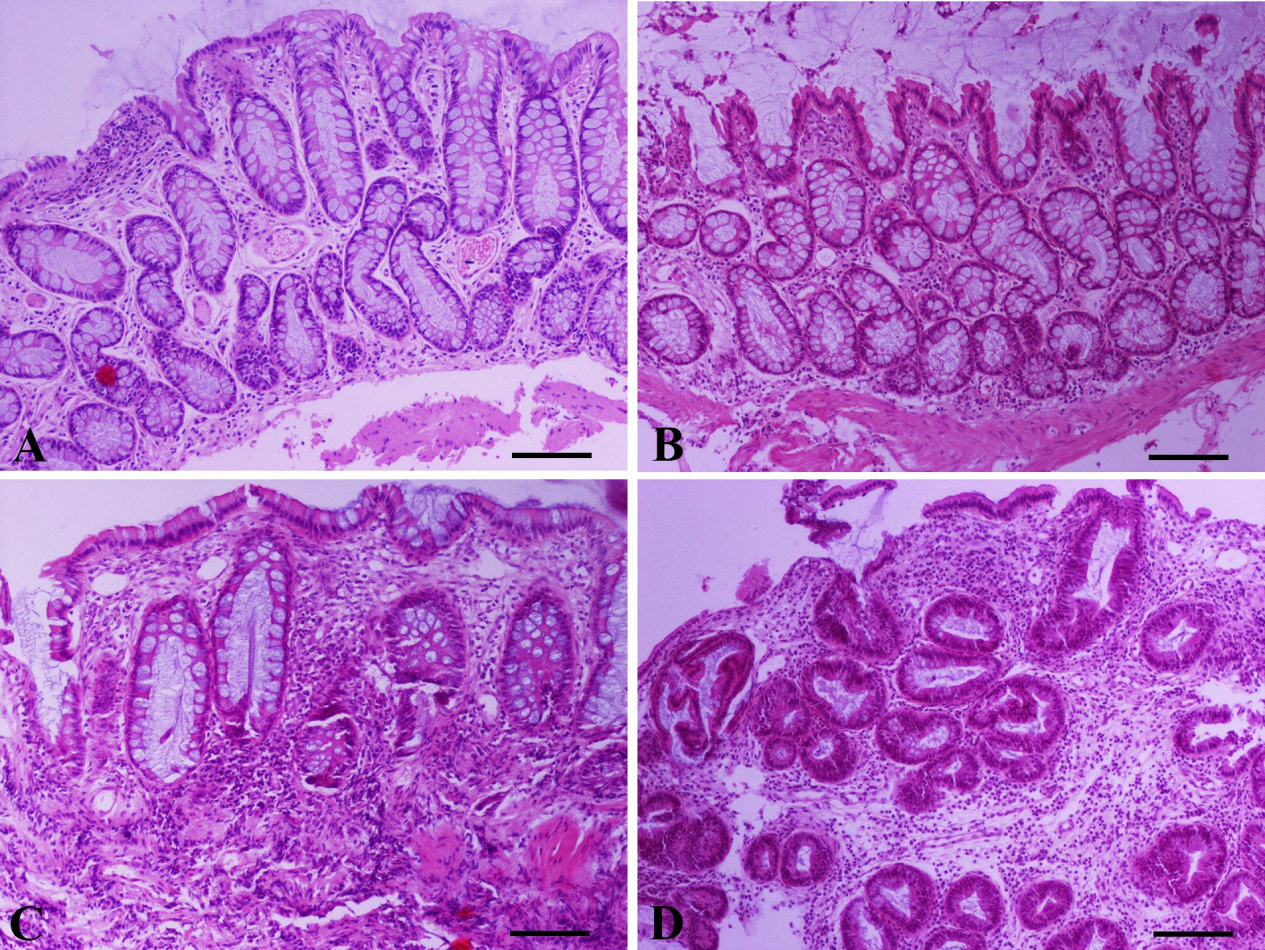
Representative photomicrographs of colonic mucosa endoscopic biopsy sections from dogs with large intestinal inflammatory bowel disease (IBD) included in the study. (Α) Mild to moderate surface epithelial injury (focal degeneration and focal loss of epithelium), mild crypt hyperplasia (basophilic and thickened lining accompanied by mild distortion), mild crypt dilatation, fibrosis among crypts and mild lymphocytic/plasmacytic inflammation. (Β) Moderate crypt hyperplasia and dilatation and irregular orientation of certain crypts. Mild fibrosis/atrophy and focally moderate lymphocytic/plasmacytic inflammation separating crypts. (C) Mild crypt hyperplasia, moderate crypt dilatation/distortion, crypt atrophy and necrosis, and moderate fibrosis. Moderate infiltration of the lamina propria by lymphocytes and to a lesser extent by plasma cells, neutrophils and macrophages. (D) Severe crypt hyperplasia with branching, dilatation and loss of orientation. Moderate fibrosis and severe infiltration of the lamina propria by lymphocytes and plasma cells. A,B,C,D: Hematoxylin and Eosin staining, bar = 100 μm.

Large bowel diarrhea, with mucus and fresh blood on the feces and tenesmus were present in all dogs with large intestinal IBD included in the study. Median CCECAI score of dogs with IBD was 7 (range = 4–11). More specifically, 9/26 dogs had a mild (range = 4–5), 10/26 dogs had a moderate (range: 6–8) and 7/26 dogs had a severe CCECAI score (range = 9–11). Median endoscopy score of dogs with IBD was 4 (range = 1–6) (Fig 1). Lymphocytes and plasma cells were the predominant cell types identified within the lamina propria inflammatory infiltrate of all 26 dogs with IBD included in this study (Fig 2 and S3 Table). Crypt hyperplasia, dilatation and distortion were also common histopathologic findings. Samples were collected from all dogs with IBD (26/26) in order to assess colonic mucosal and cytobrush samples cytokine mRNA expression. All control dogs (9/9) underwent colonoscopy in order to collect samples for histopathology and to access colonic mucosal and cytobrush samples cytokine mRNA expression.

### Cytokines relative mRNA expression in the colonic mucosa and cytobrush samples of dogs with IBD and healthy control dogs

**Fig 3:**
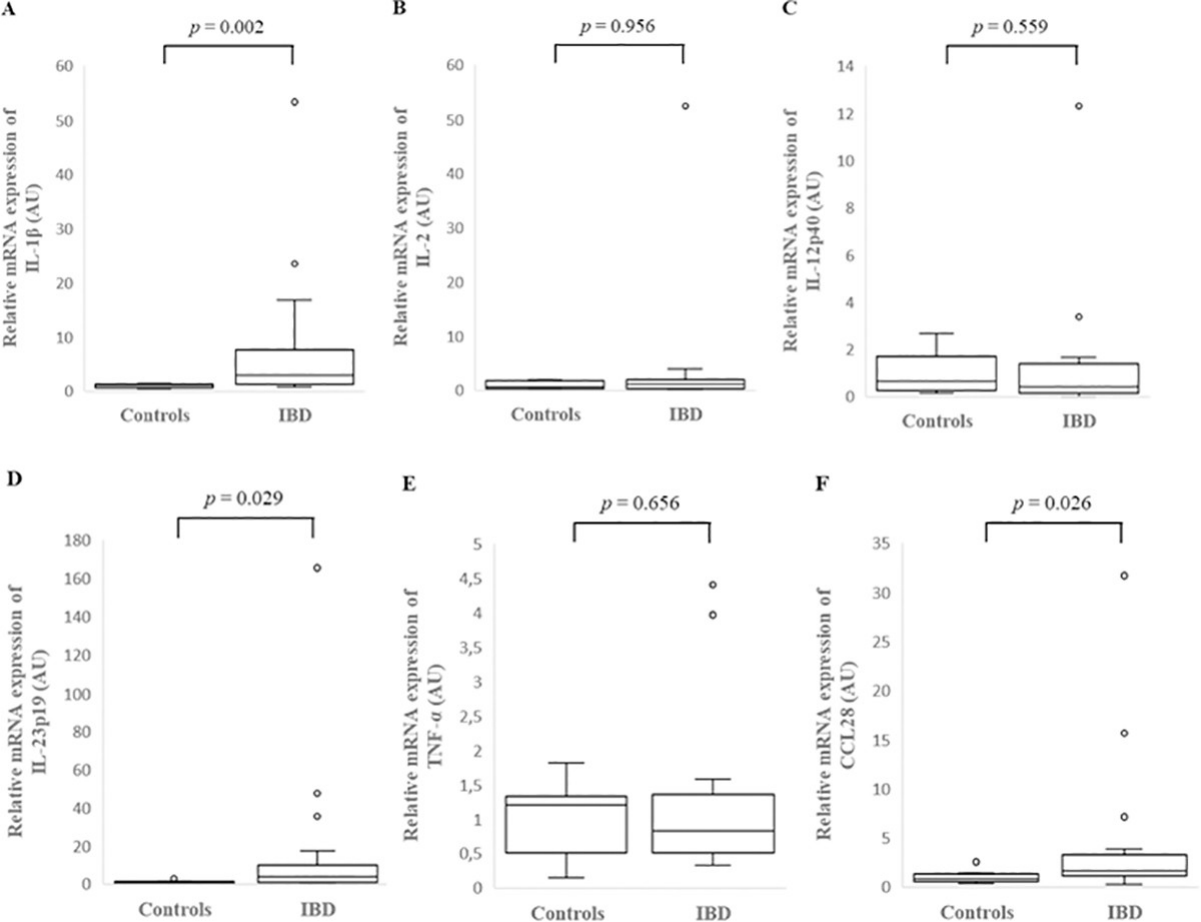
Relative mRNA expression of IL-1β (A), IL-2 (B), IL-12p40 (C), IL-23p19 (D), TNF-α (E) and CCL28 (F) in the colonic mucosa of healthy control dogs and dogs with inflammatory bowel disease (IBD). Data is presented as median with 25^th^ and 75^th^ quartiles in each box plot. The whiskers indicate the highest and lowest data within 1.5 times the lengths of the quartiles. Statistical significance was defined as *p* < 0.05. AU: arbitrary units, IL: interleukin, TNF-α: tumor necrosis factor- alpha, CCL28: C-C motif chemokine ligand 28.

**Fig 4:**
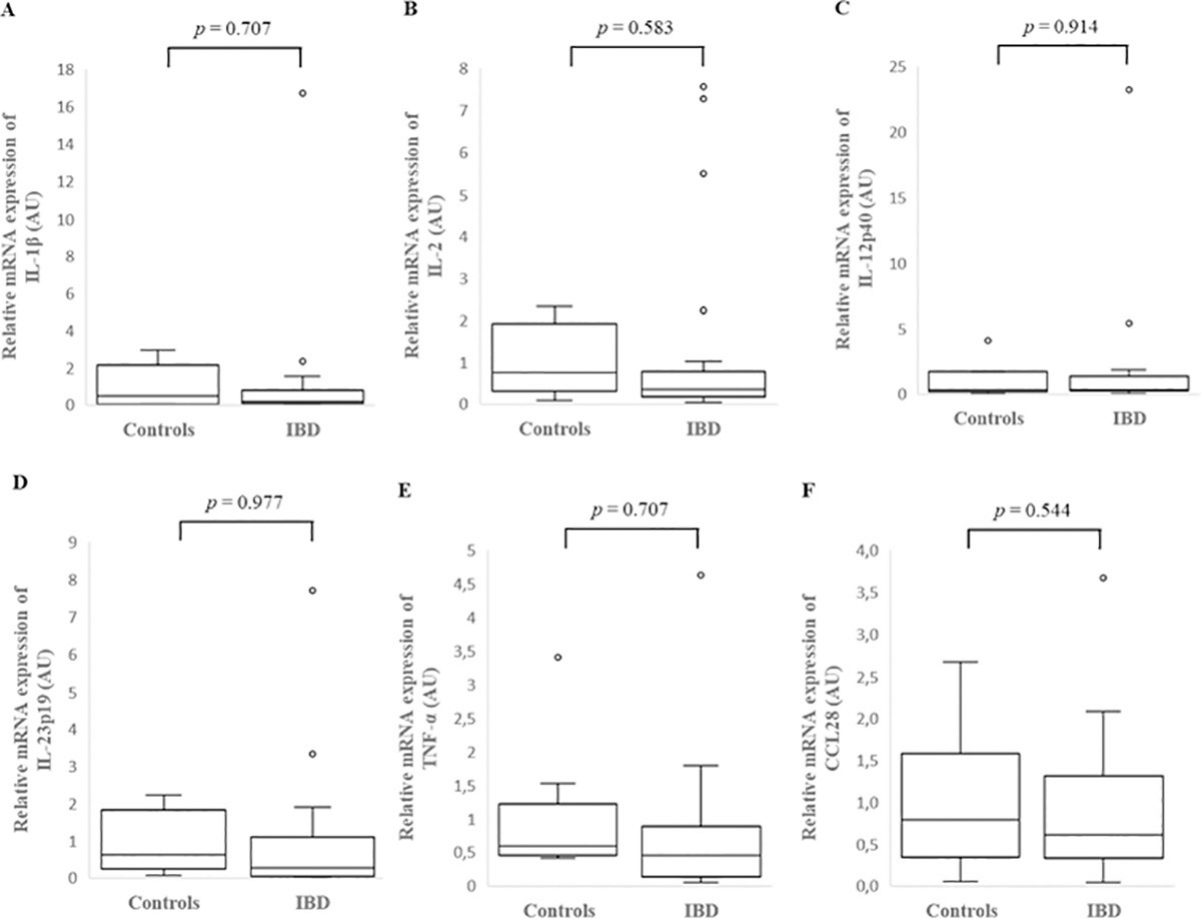
Relative mRNA expression of IL-1β (A), IL-2 (B), IL-12p40 (C), IL-23p19 (D), TNF-α (E) and CCL28 (F) in the colonic cytobrush samples of healthy control dogs and dogs with inflammatory bowel disease (IBD). Data is presented as median with 25^th^ and 75^th^ quartiles in each box plot. The whiskers indicate the highest and lowest data within 1.5 times the lengths of the quartiles. Statistical significance was defined as *p* < 0.05. AU: arbitrary units, IBD: inflammatory bowel disease, IL: interleukin, TNF-α: tumor necrosis factor- alpha, CCL28: chemokine (C-C motif) ligand 28.

IL-1β, IL-2, IL-23p19, TNF-α and CCL28 expression was assessed in the colonic mucosa of 26 dogs with IBD and 9 healthy controls and in the colonic cytobrush samples of 25 dogs with IBD and 9 healthy controls, while IL-12p40 expression was assessed in the colonic mucosa of 16 dogs with IBD and 9 healthy controls and in the colonic cytobrush samples of 15 dogs with IBD and 7 healthy controls due to the limited availability of samples. IL-1β, IL-2, IL-12p40, IL-23p19, TNF-α and CCL28 mRNA was detected in the colonic mucosa and cytobrush samples of all dogs with IBD and healthy control dogs studied. In the colonic mucosa there was a significantly increased relative mRNA expression of IL-1β (*p* = 0.002), IL-23p19 (*p* = 0.029) and CCL28 (*p* = 0.026) of dogs with IBD compared to controls (Fig 3, S4 Table). However, there was no significant difference between healthy control dogs and dogs with IBD in relative mRNA expression of IL-2 (*p* = 0.956), IL-12p40 (*p* = 0.559) and TNF-α (*p* = 0.656) (Fig 3, S4 Table). In the colonic cytobrush samples, there was no significant difference in individual cytokine mRNA expression between dogs with IBD and healthy controls (Fig 4, S4 Table).

### Cytokines relative mRNA expression in the colonic mucosa of IBD dogs with low or high CCECAI score

**Fig 5:**
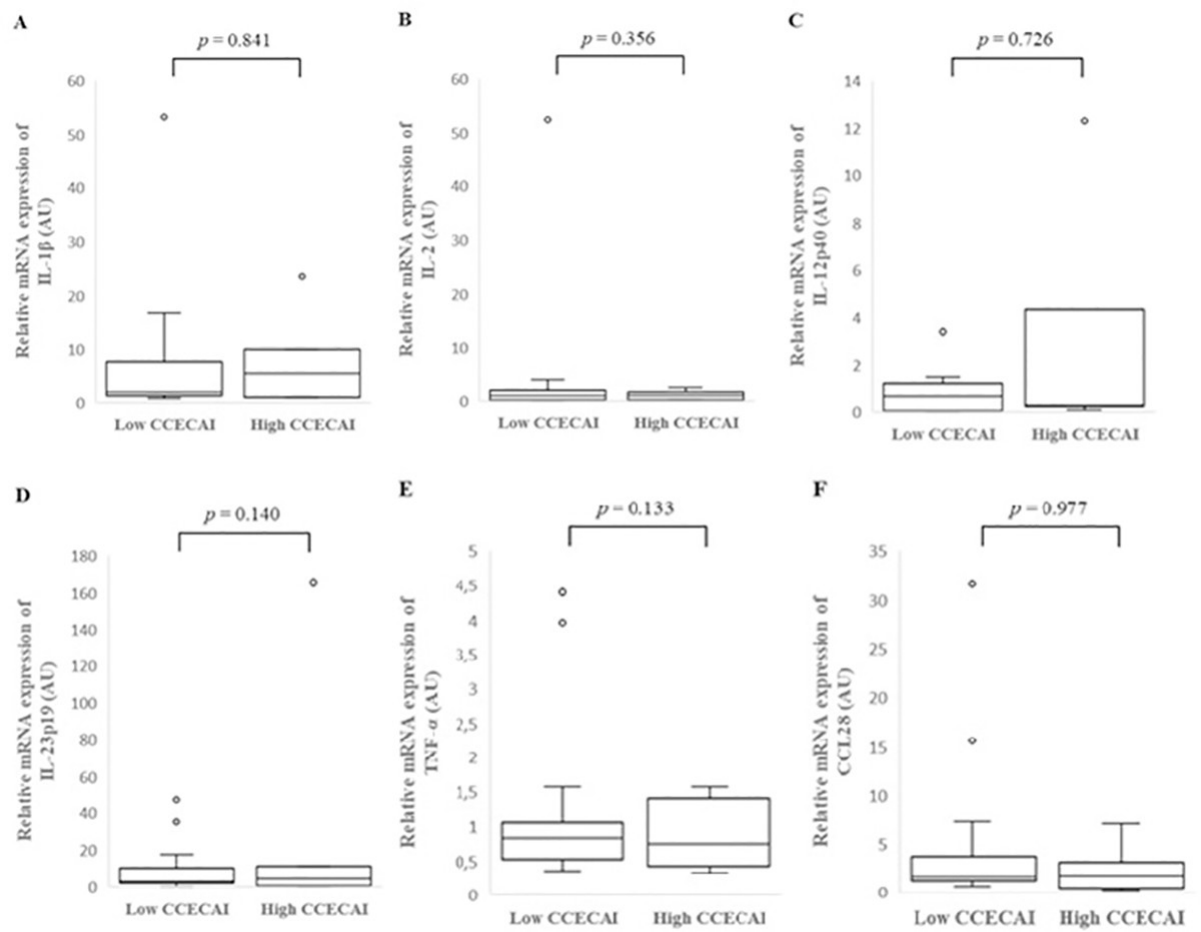
Relative mRNA expression of IL-1β (A), IL-2 (B), IL-12p40 (C), IL-23p19 (D), TNF-α (E) and CCL28 (F) in the colonic mucosa of low and high CCECAI score dogs with inflammatory bowel disease (IBD). Data is presented as median with 25^th^ and 75^th^ quartiles in each box plot. The whiskers indicate the highest and lowest data within 1.5 times the lengths of the quartiles. Statistical significance was defined as *p* < 0.05. AU: arbitrary units, IBD: inflammatory bowel disease, IL: interleukin, TNF-α: tumor necrosis factor- alpha, CCL28: chemokine (C-C motif) ligand 28.

There was no significant difference in individual cytokine mRNA expression between the colonic mucosa of dogs with low or high CCECAI score (Fig 5, S4 Table).

### Correlations

There was no significant correlation between colonic mucosal and cytobrush samples cytokine relative mRNA expression (IL-1β: *r(25)* = 0.349, *p* = 0.087; IL-2: *r(25)* = 0.330, *p* = 0.107; IL-12p40: *r(15)* = 0.048, *p* = 0.836; IL-23p19: *r(25)* = 0.390, *p* = 0.054; TNF-α: *r(25)* = 0.380, *p* = 0.061; CCL28: *r(25)* = 0.297, *p* = 0.149).

**Fig 6:**
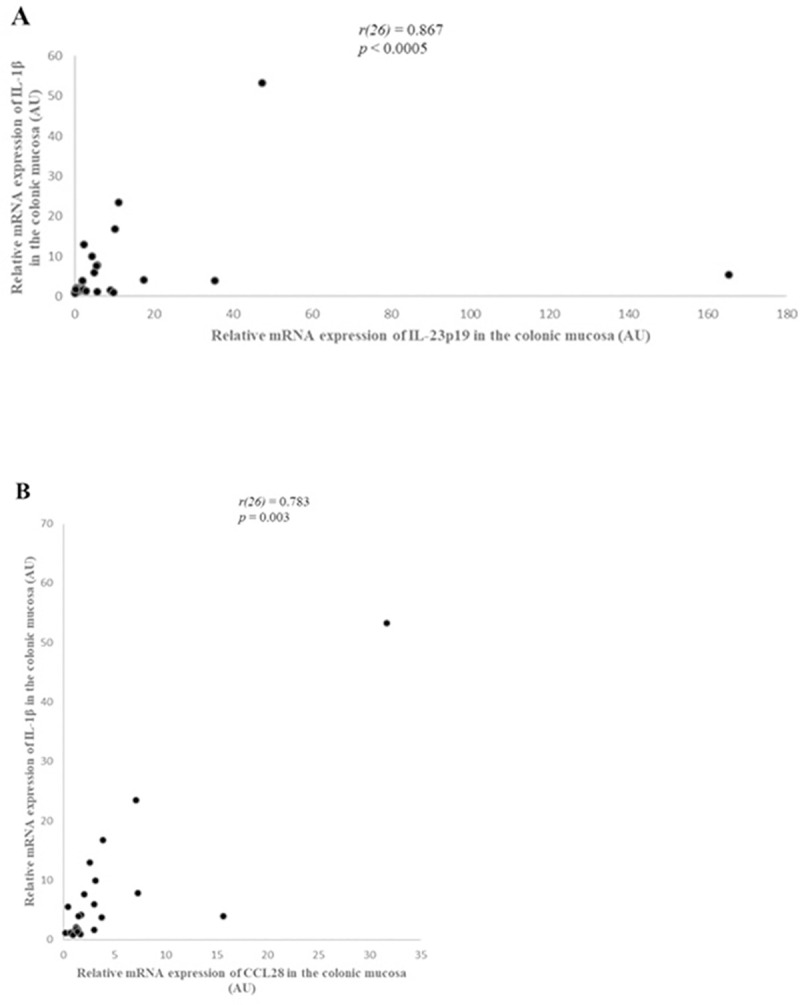
Correlation between IL-1β and IL-23p19 (A) and CCL28 (B) relative mRNA expression in the colonic mucosa of dogs with large intestinal inflammatory bowel disease (IBD) (n = 26), as determined by Spearman’s rank correlation. Statistical significance was defined as *p* < 0.05. AU: arbitrary units, CCL28: C-C motif chemokine ligand 28, IL: interleukin, *r*: Spearman’s rank correlation coefficient.

There was a strong, statistically significant positive correlation between IL-1β and CCL28 mRNA expression (*r*(26) = 0.783, *p* = 0.003) (Fig 6). In addition, there was a strong, statistically significant positive correlation between IL-1β and IL-23p19 mRNA expression (*r*(26) = 0.867, *p* < 0.0005) (Fig 6).

Correlations of cytokine relative mRNA expression in the colonic mucosa with CCECAI, colonoscopy score and histopathologic score of dogs with IBD showed that CCL28 relative mRNA expression had a moderate, statistically significant positive correlation with crypt dilation and distortion score (subscore of the WSAVA index) (*r*(26) = 0.482, *p* = 0.013) (S1 Fig). Also, IL-2 mRNA expression had a moderate, statistically significant positive correlation with surface epithelial injury score (subscore of the WSAVA index) (*r*(26) = 0.467, *p* = 0.016) (S1 Fig).

Moreover, there was a moderate, statistically significant positive correlation of the CCECAI score with the colonoscopy score (*r*(26) = 0.434, *p* = 0.027) (S2 Fig), as well as a weak, statistically significant positive correlation of the CCECAI score with the lamina propria’s neutrophils score (subscore of the WSAVA index) (*r*(26) = 0.388, *p* = 0.049) (S1 Fig). In addition, there was a moderate, statistically significant positive correlation of the CCECAI score (*r*(26) = 0.540, *p* = 0.004), of the lamina propria’s lymphocytes and plasma cells score (*r*(26) = 0.510, *p* = 0.008) and of lamina propria’s neutrophils score (*r*(26) = 0.428, *p* = 0.029) with the colonic mucosal fibrosis score (subscores of the WSAVA index) (S1 Fig).

## Discussion

This study evaluated the mRNA expression of IL-1β, IL-2, IL-12p40, IL-23p19, TNF-α and CCL28 in the colonic mucosa and cytobrush samples of dogs with IBD and healthy controls. A significantly increased relative mRNA expression of IL-1β, IL-23p19 and CCL28 in the colonic mucosa of dogs with IBD compared to healthy controls was identified. However, none of the cytokines showed significantly different relative mRNA expression in the colonic mucosa between dogs with low and high CCECAI score. In addition, none of the cytokines exhibited significantly different mRNA expression in the colonic cytobrush samples between dogs with IBD and healthy controls. The novelty of this study consists of two points. Cytokines mRNA expression was assessed in the colonic mucosa and cytobrush samples in a large number of dogs with large intestinal IBD using a quantitative real-time PCR method, and the correlations between cytokines mRNA expression and disease activity, histopathologic as well as endoscopic score were also studied.

IL-1β is a proinflammatory cytokine with a wide range of systemic and local effects associated with innate immunity and host responses to microbial invasion and tissue injury. Increased mucosal expression and production of IL-1ß has been found in Crohn’s disease and ulcerative colitis, as well as in animal models of colitis [35–39]. IL-1β has also been implicated in canine IBD pathogenesis [20–22]. Its expression has been examined in the duodenal, and to a lesser extent in the colonic mucosa of dogs with IBD [4, 6, 21, 40]. In contrast to previous studies, we found an increased IL-1β mRNA expression in the colonic mucosa of dogs with IBD. In a recent study, where a quantitative real-time PCR method was also used, no differences were seen in mRNA expression of IL-1β in dogs with large intestinal IBD as compared to healthy dogs [6]. However, only a small number of dogs with IBD was included in the latter study (n = 6). Previous studies in canine large intestinal IBD have found increased mRNA expression of toll-like receptors (TLR)2, 4 and 9 and decreased expression of TLR5, as well as increased mRNA expression of nucleotide binding oligomerization domain (NOD)2 and activity of nuclear factor-κB (NF-κB) [41–43]. These findings support the increased mRNA expression of IL-1β in the colonic mucosa observed in our study, as TLRs and NOD2 are receptors implicated in the activation of the intracellular inflammasome cascade, ultimately leading to the up-regulation of IL-1β. IL-1-β mRNA expression levels are not reliable to predict IL-1β protein expression, as IL-1β is produced through the intracellular inflammasome cascade as a pro-peptide (pro-IL-1β) intracellularly [44]. Pro-IL-1β is cleaved by the pro-inflammatory protease caspase-1, before it is secreted as the active IL-1β [44]. Recent studies have investigated IL-1β protein levels in the intestinal and colonic mucosa in canine IBD, using enzyme-linked immunosorbent assay (ELISA) or immunohistochemistry (IHC) [20–24]. Increased IL-1β protein levels in the duodenal mucosa of dogs with IBD has been reported repeatedly. With regards to the colonic mucosa of dogs with IBD, Ogawa et al. reported higher but not significantly different IL-1β protein expression compared to healthy control dogs [45]. Even though IL-1β mRNA expression was not investigated in that study, the aforementioned finding may suggest that there are differences in the pathogenesis of small and large intestinal IBD. In human IBD, elevations of IL-1β levels are associated with increased disease severity [18, 19]. In canine IBD, findings regarding the correlation of IL-1β mRNA or protein levels with disease severity are controversial [6, 20, 21, 40, 45]. In our study there was no significant correlation between the relative IL-1β mRNA expression in the colonic mucosa and the CCECAI, colonoscopy or the histopathologic parameters score in dogs with large intestinal IBD. IL-1β relative mRNA expression in the colonic mucosa of dogs with large intestinal IBD and high CCECAI score (≥9) was increased, compared to those with low CCECAI score; however, it did not reach statistical significance. In a previous study by Okanishi et al., using a quantitative real-time PCR method, no significant correlation between IL-1β mRNA expression in the duodenal mucosa and CIBDAI score in dogs with IBD was identified [40]. However, in the same study dogs with IBD and control dogs did not have significantly different IL-1β mRNA expression in the duodenal mucosa. Controversial findings regarding the correlation of IL-1β protein levels and disease severity in canine IBD have also been reported. More specifically, Hawes et al. reported a moderate positive correlation between IL-1β protein expression in the duodenum mucosa and CCECAI score in dogs with IBD [20]. Conversely, in a recent study examing the protein levels of IL-1β and IL-1 receptor antagonist (IL-1Ra) in the colonic mucosa of dogs with IBD, did not observe a significant correlation between the IL-1β:IL-1Ra ratio and CCECAI score [22]. Furthermore, Maeda et al. examined the mRNA expression and protein levels of IL-1β and IL-1Ra in the duodenal mucosa of dogs with IBD and observed only a negative correlation between the CCECAI score and the προτειν IL-1Ra:IL-1β ratio [21]. As a result, IL-1β relationship with disease severity requires further investigation. In conclusion, despite the ambiguous results between studies, that could be possibly attributed to the different study designs such as the cases included (small and/or large intestinal IBD cases), sample size and methodology used, IL-1β appears to be involved in canine IBD pathogenesis.

IL-23 is produced by macrophages and dendritic cells, induces Th17 cell differentiation [26, 46]. In canine IBD, there are limited data regarding IL-23p19 expression. In our study, as Tamura et al. previously reported, IL-23p19 relative mRNA expression in the colonic mucosa was increased in dogs with IBD compared to healthy controls [6]. Moreover, in our study IL-1β mRNA expression was increased and there was a strong positive correlation between IL-1β and IL-23p19 expression in the colonic mucosa of dogs with IBD. In humans, IL-23p19 has been associated with IBD pathogenesis and disease severity [28, 34, 47, 48]. Furthermore, in vitro studies have shown a role of IL-1β, in conjunction with IL-6 and IL-23, in promoting the Th17 cells differentiation [49–54]. In addition, previous studies in mice and humans have found that IL-1β participates in Th17 differentiation, while autoinflammatory diseases characterized by increased levels of IL-1β and are associated with a marked Th17 signature [55–57]. In humans, Crohn’s disease is primarily associated with Th1 immune responses and ulcerative colitis predominantly shows a characteristic atypical Th2 cytokine pattern [58]. However, in recent studies is reported that the inflamed intestinal regions in both Crohn’s disease and ulcerative colitis are significantly infiltrated with Th17 cells with unique cytokine responses [59–61]. Regarding canine IBD, a few studies have evaluated the Th17 subset (gene and/or protein expression of IL-17A, IL-22, IL-10, IFN-γ, TGF-β, IL-1β, IL-8) in the duodenal mucosa of dogs with IBD and did not find any evidence for the involvement of Th 17 cytokines in disease pathogenesis [10, 24, 62]. The increased mRNA expression of IL-23p19 in the colonic mucosa of dogs with IBD found in this study, could suggest that a Th17 type response may occur. However, there was no significant correlation between IL-23p19 relative mRNA expression and clinical, endoscopic or histopathologic disease severity scores including the lamina propria infiltration by macrophages, possibly reflecting the different etiopathogenetic mechanisms between human and canine IBD.

CCL28 is a member of the C-C chemokine subfamily and is expressed in the epithelial cells of different mucosal sites, including colon and to a substantially lesser extent in the small intestine [63, 64]. CCL28 has an important role in mucosal immunity as a chemoattractant for cells expressing CCR10 and is also a broad-spectrum antimicrobial protein. More specifically, it induces the migration of IgA-secreting plasma cells and T cells mediated by its receptor CCR10 and has a broad spectrum antimicrobial activity against Gram-positive and Gram-negative bacteria and fungi [65, 66]. CCL28 mRNA expression is markedly increased in the epithelium of pathologically inflamed compared with normal human colon [65]. Furthermore, increased CCL28 levels have been reported in the colonic mucosa and serum of ulcerative colitis patients, using immunohistochemistry and enzyme-linked immunosorbent assay, respectively [67]. To our knowledge there are no studies evaluating CCL28 expression in the colonic mucosa of dogs with IBD. Maeda et al. studied the mRNA expression of various chemokines and chemokine receptors, including CCL28, in small intestinal IBD [29]. Similar to our findings, CCL28 expression was increased in the duodenal mucosa of dogs with IBD compared to healthy controls, and it could be concluded that CCL28 has an important role in the pathogenesis of canine IBD, regardless the GI segment (small or large intestine) affected. CCL28 production is upregulated by proinflammatory stimuli including microbial pathogens, microbial products, and proinflammatory cytokines such as TNF-a and IL-1 [65, 68, 69]. In human colon, CCL28 expression is induced by IL-1α, bacterial flagellin and *n*-butyrate but not by TNF-α [65]. In our study, mRNA transcripts of IL-1β and CCL28 were up-regulated in the colonic mucosa of dogs with IBD, and there was a strong positive correlation between their expression. To the contrary, TNF-α mRNA expression in the colonic mucosa did not show any significant differences between dogs with IBD and controls. So, IL-1β, and not TNF-α, could be an important inducer for the over-expression of CCL28 in the colonic mucosa of dogs with IBD. However, at this time there is no direct evidence to support this hypothesis and further studies are needed to identify inducing factors of CCL28 production in the colonic mucosa of dogs with IBD. In this context, it is also worth mentioning that IL-1β is activated within the context of the inflammasome cascade, and that mRNA levels include the measurement of pro-IL1β, which still needs to be cleaved by caspase before it is secreted in its active form, IL-1β. Therefore, mRNA levels of this cytokines may be even less correlated with actual protein expression levels than is the case with other cytokines. Ideally, therefore, Il-1β should always be measured as expressed protein.

According to the results of the present study, IL-2 and TNF-α did not show a significantly different relative mRNA expression in the colonic mucosa of 26 dogs with IBD compared to healthy controls. In previous reports, their expression in the intestinal and the colonic mucosa in canine IBD measured by semi-quantitative methods was variable [4, 7, 9, 25]. In a more recent study by Tamura et al., using a quantitative real-time PCR method, a significant different expression of IL-2 and TNF-α in the colonic mucosa of 6 dogs with IBD compared to healthy controls was not identified [6]. Both semi-quantitative and quantitative methods, as well as the greater number of dogs included in our study did not yield any significantly different mRNA expression of these cytokines and possibly demonstrate that canine large intestinal IBD is not characterized by a Th1 polarization. Consequently, future studies targeting cytokine mRNA expression profiles in canine IBD should not focus on these cytokines.

Another finding of this study was the moderate positive correlation of IL-2 mRNA expression in the colonic mucosa with surface epithelial injury score. There are limited studies regarding the role of intestinal epithelial cells in canine IBD. Various growth factors, cytokines, and trefoil enhance restitution or proliferation of intestinal epithelial cells [70]. A previous study showed that IL-2 enhances epithelial cell migration in an in vitro wounding model and supports the hypothesis that it may play an important role in epithelial cell restitution following various forms of intestinal injury, including inflammation [71]. This finding suggests that IL-2 role in IBD may not be limited in stimulating inflammation, but also in preserving the integrity of the intestinal epithelium (mucosal homeostasis) and could explain the correlation found in our study.

mRNA of all cytokines studied was also detected in the colonic cytobrush samples of healthy controls of the present study, and no significant difference was detected between the two groups. Furthermore, correlation of cytokines relative mRNA expression between colonic mucosa and cytobrush samples was not significant in the IBD group. This finding could be due to the amount of RNA extracted from the cytobrush samples, which is low, compared to that extracted from the colonic mucosa biopsies. The low number of cells sampled and the uncertainty of being representative of the inflammatory process of the intestinal/colonic mucosa seen in canine IBD probably affected the results in our case indicating that there are limitations of mRNA expression analysis using cytobrush samples.

In our study, there was a moderately positive correlation of the CCECAI score with the endoscopic score, suggesting that the clinical signs may reflect the lesions seen during endoscopy, and they may serve as a predictive index concerning their severity. To the best of our knowledge, only one study has evaluated the relationship of the clinical disease activity with the endoscopic score in canine IBD at the time of diagnosis [2]. There is a discrepancy between the results of that study and our findings, as a statistically significant correlation between CIBDAI and endoscopy score was not identified in the previous study. The differences could be mainly attributed to the fact that our study focused on large intestinal IBD, while the other study included dogs suffering from various chronic enteropathies. Another possible explanation could be the different evaluation indices used in the two studies.

Finally, we studied the correlation between histopathologic parameters score with CCECAI and colonoscopy score, as well as the correlation between the cellular infiltrates and architectural changes in the colonic mucosa. In canine IBD, several studies have tried unsuccessfully to correlate the severity of the histopathologic changes with clinical disease severity (or clinical signs), serum biomarkers or even response to treatment [2, 72–75]. The results of our study confirm the current knowledge, that histopathologic findings do not reflect completely neither the severity of clinical presentation nor the macroscopic lesions identified on colonoscopy. However, in our study, there was a weak and a moderate statistically significant positive correlation of the CCECAI score with the lamina propria neutrophils and the colonic mucosal fibrosis and atrophy, respectively. Neutrophils are destructive cells and their infiltration in various tissues can lead to severe architectural changes [76]. Considering this fact and taking into account that these cells are seen sparsely and usually in limited numbers in canine IBD, may explain the weak and positive correlation between CCECAI score and lamina propria neutrophils found in our study. The latter finding is consistent with a recent study by Allenspach et al., in which there was a moderate, statistically significant positive correlation between the CCECAI and the colonic mucosal fibrosis and atrophy score [16]. Fibrosis is a consequence of local chronic inflammation that develops only in the inflamed intestinal segments, and is characterized by excessive extracellular matrix protein deposition in the intestinal wall layers [77–80]. In canine IBD, fibrosis does not result to intestinal stenosis; however, there is lack of data regarding its clinical impact and pathophysiology. Fibrosis in UC could be responsible for diarrhea and tenesmus, even in the absence of active inflammation [81]. After further analysis, a moderate, statistically significant positive correlation was found between fibrosis and atrophy, and the number of lymphocytes and plasma cells, and neutrophils infiltrating the lamina propria of the colonic mucosa, indicating perhaps that the severity of cellular infiltration correlates with the severity of architectural changes. Regarding the relationship of cytokines expression with mucosal fibrosis, it has been reported that IL-1β and TNF-α have a potential role on intestinal fibrosis in human IBD [82, 83]. However, none of the cytokines studied, including IL-1β and TNF-α, had a significant correlation with the mucosal fibrosis score in our study.

This study had some limitations. Our strict study inclusion criteria (disease free animals with normal diagnostic tests) resulted in a small population of healthy control dogs, that were younger and had discrepancy in genetic background compared to case population. The majority of control dogs were of mixed-breed origin and were considered a better choice versus age-matched, research reared Beagles which might have evidence of intestinal disease [84]. It is important to acknowledge that environmental factors (age, genetic background and dietary history) may have possibly biased our results. Another potential limitation is the small number of cytokines examined. The aim of our study was to examine the expression of a cytokine panel and investigate their correlation with clinical, endoscopic and histopathologic scores. Cytokines selection was based on their role in mucosal inflammation, disease pathogenesis and severity. However, our study revealed some interesting findings such as the increased expression of IL-1β and IL-23p19 in the colonic mucosa, suggesting that a Th17 type response may occur in large intestinal IBD and highlighting the need for further studies including Th17 cytokines expression analysis.

## Conclusions

In conclusion, our study indicates that the colonic mucosal IL-1β, IL-23p19 and CCL28 mRNA expression were significantly increased in the mucosa of dogs with large intestinal IBD in comparison to healthy dogs. IL-1β, IL-23p19 and CCL28 may play a role in canine large intestinal IBD pathogenesis, but their expression did not correlate with clinical disease and/or endoscopic activity score. These results suggest that cytokines mRNA expression levels analyses may not be a vital marker of canine large intestinal IBD, even though they offer further insights in disease pathogenesis. Cytokines expression in the colonic cytobrush samples did not reveal useful information regarding canine IBD. Finally, clinical disease activity (CCECAI) score correlated with endoscopic activity score and fibrosis and atrophy scores in dogs with large intestinal IBD.

## Supporting information

S1 Fig Correlation between IL-2 relative mRNA expression in the colonic mucosa and surface epithelial injury score (subscore of the WSAVA index) (A), CCL28 relative mRNA expression in the colonic mucosa and crypt dilation and distortion score (subscore of the WSAVA index) (B), CCECAI score and the lamina propria’s neutrophils score (subscore of the WSAVA index) (C), and of the colonic mucosal fibrosis score (subscores of the WSAVA index) and CCECAI score (D), lamina propria’s lymphocytes and plasma cells score (E) and lamina propria’s neutrophils score (F) of dogs with large intestinal inflammatory bowel disease (IBD) (n = 26) as determined by Spearman’s rank correlation. Statistical significance was defined as *p* < 0.05. AU: arbitrary units, CCL28: chemokine (C-C motif) ligand 28, CCECAI: canine chronic enteropathy clinical activity index, IL: interleukin, LP: lamina propria, *r*: Spearman’s rank correlation coefficient.
(DOCX)

S2 Fig Correlation between colonoscopy and CCECAI score of dogs with large intestinal inflammatory bowel disease (IBD) (n = 26) as determined by Spearman’s rank correlation. Statistical significance was defined as *p* < 0.05. CCECAI = canine chronic enteropathy clinical activity index, *r* = Spearman’s rank correlation coefficient.
(DOCX)

S1 Table Epidemiologic data, canine chronic enteropathy clinical activity index (CCECAI) score and colonoscopy score of 26 dogs with inflammatory bowel disease (IBD). M: male, Mn: male neutered, F: female, Fs: female spayed, WHWT: West Highland White Terrier, GSD: German Shepherd Dog.
(DOCX)

S2 Table Epidemiologic data of 9 healthy dogs. M: male, F: female, Fs: female spayed, GSD: German Shepherd Dog.
(DOCX)

S3 Table Histopathologic scores of 26 dogs with inflammatory large bowel disease. (DOCX)

S4 Table Relative mRNA expression of IL-1β, IL-2, IL-12p40, IL-23p19, TNF-α and CCL28 in the colonic mucosa and cytobrush samples of dogs with inflammatory bowel disease (IBD) (n = 26) and healthy control dogs (n = 9). Relative expression of a gene in each sample was calculated as arbitrary units by dividing its mean normalized expression by the average level of respective measurements in control samples. IBD: inflammatory bowel disease, IL: interleukin, TNF-α: tumor necrosis factor- alpha, CCL28: chemokine (C-C motif) ligand 28, ND: not done.
(DOCX)
