# Supplementary figures and images for: Colonic mucosal and cytobrush sample cytokine mRNA expression in canine inflammatory bowel disease and their correlation with disease activity, endoscopic and histopathologic score
Source: PLoS One. 2021 Jan 20;16(1):e0245713. doi: 10.1371/journal.pone.0245713 (PMC7817028; doi:10.1371/journal.pone.0245713)

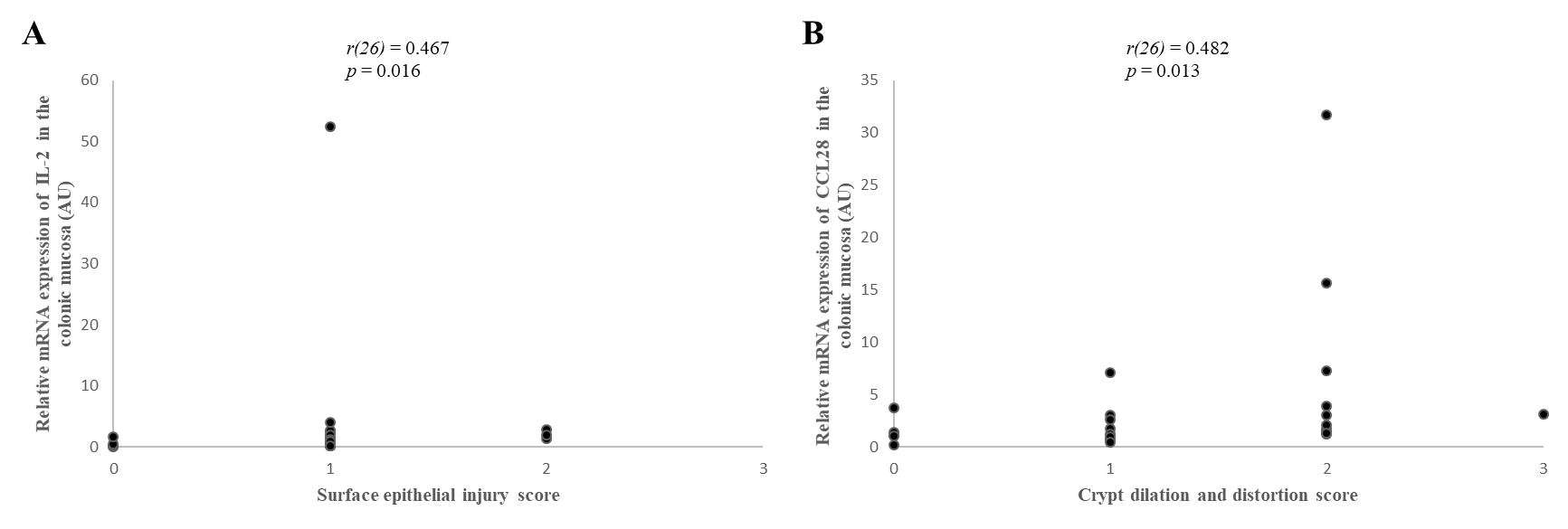


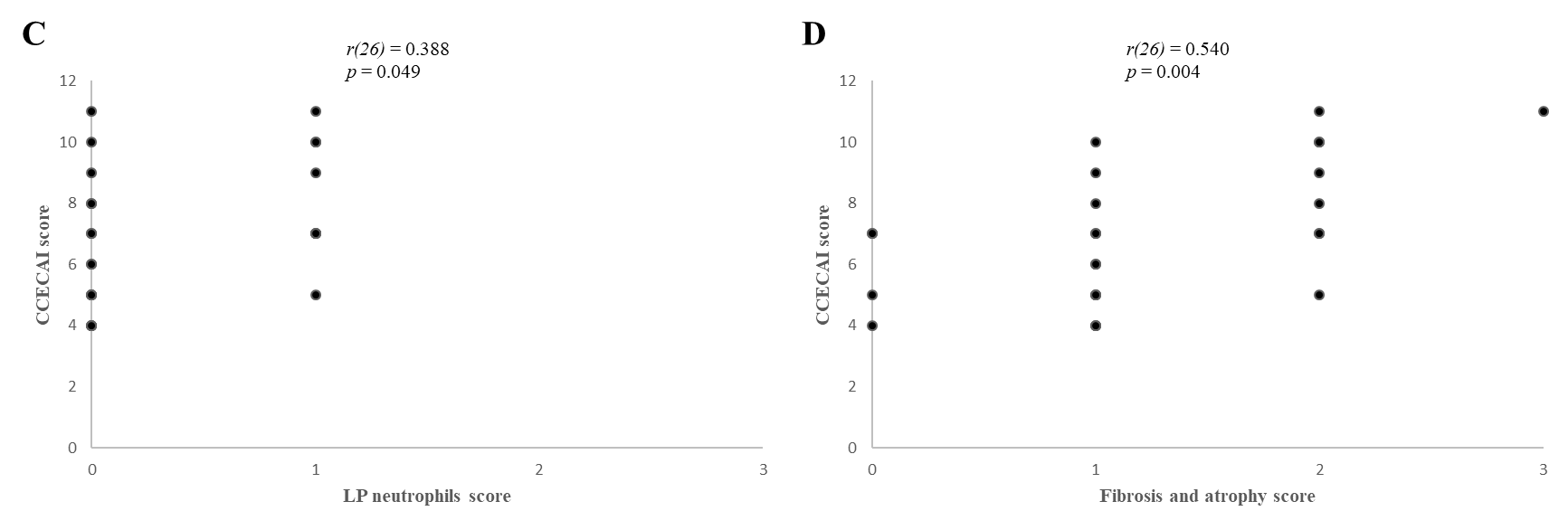


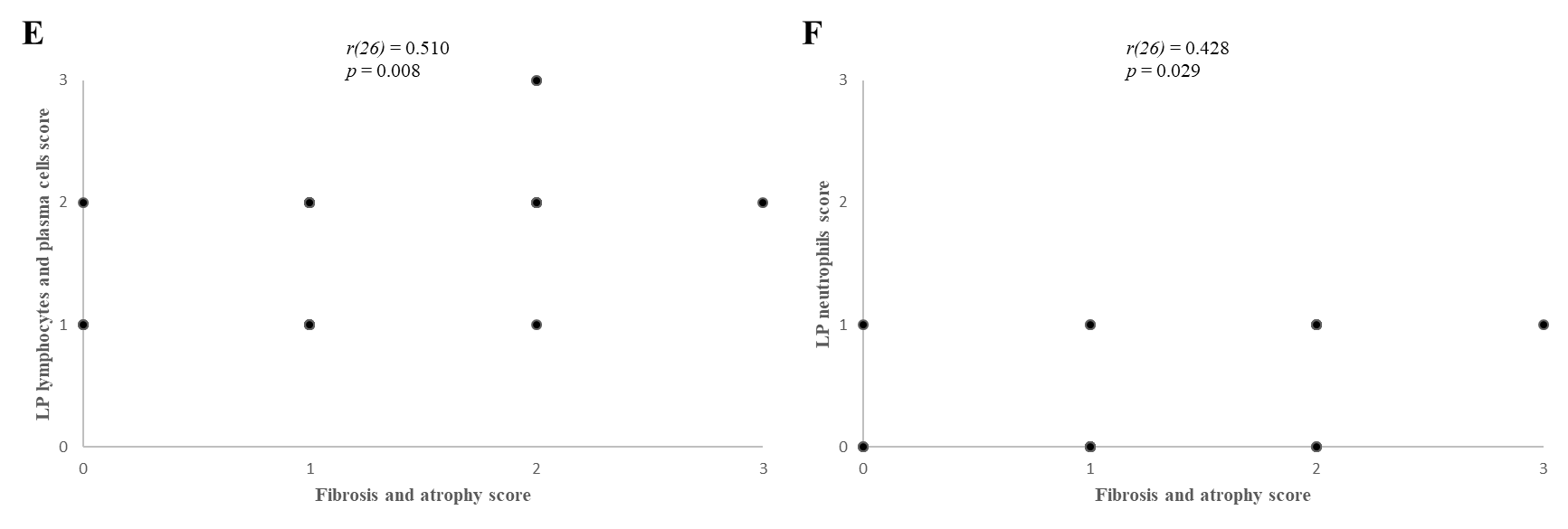

Supplement: S1 Fig — Correlation between IL-2 relative mRNA expression in the colonic mucosa and surface epithelial injury score (subscore of the WSAVA index) (A), CCL28 relative mRNA expression in the colonic mucosa and crypt dilation and distortion score (subscore of the WSAVA index) (B), CCECAI score and the lamina propria’s neutrophils score (subscore of the WSAVA index) (C), and of the colonic mucosal fibrosis score (subscores of the WSAVA index) and CCECAI score (D), lamina propria’s lymphocytes and plasma cells score (E) and lamina propria’s neutrophils score (F) of dogs with large intestinal inflammatory bowel disease (IBD) (n = 26) as determined by Spearman’s rank correlation. Statistical significance was defined as p < 0.05. AU: arbitrary units, CCL28: chemokine (C-C motif) ligand 28, CCECAI: canine chronic enteropathy clinical activity index, IL: interleukin, LP: lamina propria, r: Spearman’s rank correlation coefficient. (DOCX) [file pone.0245713.s001.docx]

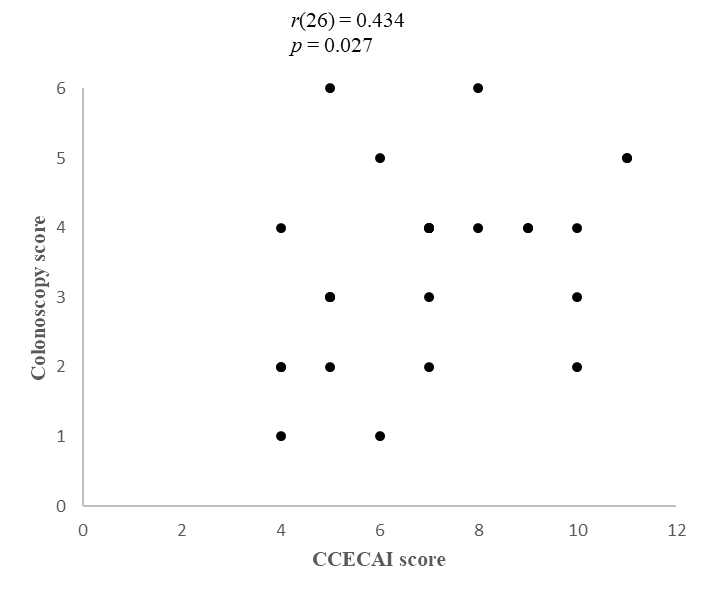

Supplement: S2 Fig — Statistical significance was defined as p < 0.05. CCECAI = canine chronic enteropathy clinical activity index, r = Spearman’s rank correlation coefficient. (DOCX) [file pone.0245713.s002.docx]
