# Supplementary material for: Colonic mucosal and cytobrush sample cytokine mRNA expression in canine inflammatory bowel disease and their correlation with disease activity, endoscopic and histopathologic score
Source: PLoS One. 2021 Jan 20;16(1):e0245713. doi: 10.1371/journal.pone.0245713 (PMC7817028; doi:10.1371/journal.pone.0245713)
Supplement: S1 Table — M: male, Mn: male neutered, F: female, Fs: female spayed, WHWT: West Highland White Terrier, GSD: German Shepherd Dog. (DOCX) [file pone.0245713.s003.docx]

| **Case No.** | **Breed** | **Sex** | **Body weight (kg)** | **Age (years)** | **CCECAI score** | **Quantitative colonoscopy score** |
| --- | --- | --- | --- | --- | --- | --- |
| IBD1 | Mongrel | M | 7.6 | 2.5 | 7 | 3 |
| IBD2 | Chihuahua | M | 1.72 | 3 | 11 | 5 |
| IBD3 | American Pitbull | F | 24 | 1.2 | 5 | 3 |
| IBD4 | Chihuahua | Fs | 2.5 | 6 | 8 | 6 |
| IBD5 | Miniature Pinscher | M | 7.5 | 9.5 | 8 | 4 |
| IBD6 | Mongrel | M | 8 | 5.5 | 7 | 4 |
| IBD7 | Mongrel | Fs | 25 | 12 | 5 | 2 |
| IBD8 | Miniature Pinscher | M | 1.85 | 7 | 10 | 4 |
| IBD9 | Labrador Retriever | Fs | 21.2 | 1.5 | 9 | 4 |
| IBD10 | WHWT | M | 8 | 3 | 5 | 6 |
| IBD11 | Mongrel | Fs | 25.3 | 6 | 7 | 4 |
| IBD12 | Mongrel | M | 27.9 | 4 | 6 | 5 |
| IBD13 | Mongrel | F | 7.15 | 2.5 | 7 | 2 |
| IBD14 | Maltese | M | 1.95 | 11 | 9 | 4 |
| IBD15 | American Pitbull | M | 25.5 | 13 | 4 | 4 |
| IBD16 | Mongrel | M | 9.5 | 8 | 4 | 2 |
| IBD17 | Pug | Fs | 7.95 | 12.5 | 7 | 4 |
| IBD18 | American Pitbull | M | 26.8 | 3.5 | 6 | 1 |
| IBD19 | WHWT | M | 8.5 | 11 | 4 | 2 |
| IBD20 | Mongrel | Fs | 23 | 7 | 5 | 3 |
| IBD21 | Yorkshire Terrier | M | 3.4 | 8.5 | 7 | 4 |
| IBD22 | Maltese | Fs | 4.25 | 7 | 10 | 3 |
| IBD23 | GSD | Mn | 36.5 | 1.5 | 5 | 3 |
| IBD24 | Mongrel | Fs | 22.1 | 6 | 4 | 1 |
| IBD25 | Yorkshire Terrier | Fs | 4.1 | 15 | 11 | 5 |
| IBD26 | Bichon Frise | Fs | 7.1 | 12 | 10 | 2 |
