# Supplementary material for: Colonic mucosal and cytobrush sample cytokine mRNA expression in canine inflammatory bowel disease and their correlation with disease activity, endoscopic and histopathologic score
Source: PLoS One. 2021 Jan 20;16(1):e0245713. doi: 10.1371/journal.pone.0245713 (PMC7817028; doi:10.1371/journal.pone.0245713)
Supplement: S2 Table — M: male, F: female, Fs: female spayed, GSD: German Shepherd Dog. (DOCX) [file pone.0245713.s004.docx]

| **Control No.** | **Breed** | **Sex** | **Age (years)** | **Body weight (kg)** |
| --- | --- | --- | --- | --- |
| C1 | Golden Retriever | M | 7 | 35 |
| C2 | Mongrel | M | 2 | 32 |
| C3 | Mongrel | M | 2 | 26 |
| C4 | Mongrel | M | 2 | 29 |
| C5 | Mongrel | Fs | 2 | 24 |
| C6 | Mongrel | Fs | 5 | 26 |
| C7 | GSD | Fs | 4 | 27 |
| C8 | Mongrel | M | 2.5 | 28 |
| C9 | Mongrel | M | 2.5 | 31 |
