# Supplementary material for: Colonic mucosal and cytobrush sample cytokine mRNA expression in canine inflammatory bowel disease and their correlation with disease activity, endoscopic and histopathologic score
Source: PLoS One. 2021 Jan 20;16(1):e0245713. doi: 10.1371/journal.pone.0245713 (PMC7817028; doi:10.1371/journal.pone.0245713)
Supplement: S3 Table — (DOCX) [file pone.0245713.s005.docx]

| **Case No.** | **Surface epithelial injury** | **Crypt hyperplasia** | **Crypt dilation and distortion** | **Fibrosis and atrophy** | **Lamina propria lymphocytes/ plasma cells** | **Lamina propria neutrophils** | **Lamina propria eosinophils** | **Lamina propria macrophages** |
| --- | --- | --- | --- | --- | --- | --- | --- | --- |
| IBD1 | 1 | 3 | 0 | 2 | 3 | 1 | 0 | 0 |
| IBD2 | 1 | 2 | 0 | 2 | 2 | 0 | 0 | 1 |
| IBD3 | 1 | 1 | 1 | 1 | 1 | 0 | 0 | 0 |
| IBD4 | 0 | 2 | 1 | 1 | 1 | 0 | 1 | 1 |
| IBD5 | 0 | 2 | 2 | 2 | 1 | 0 | 0 | 1 |
| IBD6 | 1 | 1 | 0 | 1 | 2 | 1 | 0 | 1 |
| IBD7 | 1 | 1 | 0 | 1 | 2 | 0 | 0 | 0 |
| IBD8 | 2 | 3 | 3 | 2 | 3 | 1 | 0 | 1 |
| IBD9 | 1 | 1 | 1 | 1 | 1 | 1 | 0 | 1 |
| IBD10 | 2 | 2 | 2 | 2 | 2 | 1 | 0 | 1 |
| IBD11 | 0 | 2 | 2 | 0 | 1 | 1 | 0 | 0 |
| IBD12 | 1 | 2 | 1 | 1 | 2 | 0 | 0 | 1 |
| IBD13 | 2 | 2 | 1 | 2 | 2 | 1 | 0 | 1 |
| IBD14 | 1 | 3 | 2 | 2 | 2 | 0 | 0 | 0 |
| IBD15 | 0 | 2 | 1 | 1 | 2 | 0 | 0 | 0 |
| IBD16 | 1 | 2 | 2 | 0 | 1 | 0 | 0 | 0 |
| IBD17 | 1 | 2 | 1 | 0 | 1 | 0 | 0 | 1 |
| IBD18 | 1 | 2 | 2 | 1 | 1 | 0 | 0 | 1 |
| IBD19 | 1 | 2 | 2 | 1 | 2 | 0 | 0 | 1 |
| IBD20 | 1 | 1 | 1 | 0 | 2 | 0 | 0 | 0 |
| IBD21 | 1 | 2 | 2 | 1 | 1 | 0 | 0 | 0 |
| IBD22 | 1 | 2 | 1 | 1 | 2 | 0 | 1 | 1 |
| IBD23 | 1 | 2 | 2 | 1 | 2 | 0 | 0 | 0 |
| IBD24 | 1 | 2 | 2 | 1 | 1 | 0 | 0 | 1 |
| IBD25 | 0 | 1 | 2 | 3 | 2 | 1 | 0 | 1 |
| IBD26 | 1 | 2 | 0 | 2 | 2 | 1 | 0 | 0 |
