# Supplementary material for: Colonic mucosal and cytobrush sample cytokine mRNA expression in canine inflammatory bowel disease and their correlation with disease activity, endoscopic and histopathologic score
Source: PLoS One. 2021 Jan 20;16(1):e0245713. doi: 10.1371/journal.pone.0245713 (PMC7817028; doi:10.1371/journal.pone.0245713)
Supplement: S4 Table — Relative expression of a gene in each sample was calculated as arbitrary units by dividing its mean normalized expression by the average level of respective measurements in control samples. IBD: inflammatory bowel disease, IL: interleukin, TNF-α: tumor necrosis factor- alpha, CCL28: chemokine (C-C motif) ligand 28, ND: not done. (DOCX) [file pone.0245713.s006.docx]

|  | **Colonic mucosa** | | | | | | **Cytobrush samples** | | | | | |
| --- | --- | --- | --- | --- | --- | --- | --- | --- | --- | --- | --- | --- |
|  | **IL-1β** | **IL-2** | **IL-12p40** | **IL-23p19** | **TNF-α** | **CCL28** | **IL-1β** | **IL-2** | **IL-12p40** | **IL-23p19** | **TNF-α** | **CCL28** |
| **IBD1** | 1.269 | 1.010 | 0.456 | 0.807 | 0.559 | 1.174 | 0.025 | 0.122 | 0.524 | 0.030 | 0.045 | 0.440 |
| **IBD2** | 1.071 | 0.248 | 0.258 | 0.439 | 0.436 | 0.239 | 0.102 | 0.168 | 0.119 | 0.027 | 0.060 | 0.245 |
| **IBD3** | 1.595 | 1.462 | 1.467 | 8.985 | 0.851 | 3.039 | 2.318 | 7.278 | 23.221 | 7.711 | 4.625 | 0.527 |
| **IBD4** | 1.248 | 0.027 | 1.092 | 0.467 | 0.532 | 0.737 | 0.177 | 0.033 | 0.224 | 0.387 | 0.096 | 0.602 |
| **IBD5** | 2.036 | 0.163 | 0.408 | 0.405 | 0.501 | 1.258 | 0.105 | 0.114 | 0.031 | 0.060 | 0.080 | 1.254 |
| **IBD6** | 1.617 | 0.717 | 0.010 | 2.215 | 0.520 | 1.430 | 0.499 | 0.191 | ND | 0.141 | 0.917 | 0.353 |
| **IBD7** | 3.744 | 1.793 | 0.971 | 1.890 | 1.012 | 3.756 | 0.191 | 0.675 | 0.348 | 0.559 | 0.459 | 1.809 |
| **IBD8** | 9.944 | 1.377 | 12.295 | 4.451 | 1.570 | 3.150 | 0.714 | 0.212 | 1.883 | 0.030 | 0.769 | 1.114 |
| **IBD9** | 23.501 | 2.502 | 1.669 | 11.153 | 0.747 | 7.118 | 1.364 | 0.248 | 1.330 | 0.266 | 0.178 | 0.327 |
| **IBD10** | 16.736 | 2.838 | ND | 10.105 | 3.966 | 3.905 | 1.532 | 0.861 | ND | 0.483 | 1.794 | 0.901 |
| **IBD11** | 1.007 | 0.202 | ND | 9.801 | 0.853 | 1.635 | 0.193 | 0.339 | ND | 1.448 | 0.447 | 0.258 |
| **IBD12** | 1.144 | 0.484 | ND | 5.651 | 0.833 | 0.683 | 0.068 | 0.533 | ND | 3.329 | 0.833 | 0.930 |
| **IBD13** | 4.114 | 1.951 | ND | 17.537 | 1.576 | 1.754 | 0.115 | 0.034 | ND | 1.154 | 0.670 | 0.041 |
| **IBD14** | 0.950 | 0.152 | 0.267 | 0.099 | 0.334 | 1.683 | 16.695 | 0.274 | 5.422 | 1.034 | 0.912 | 0.076 |
| **IBD15** | 1.713 | 0.480 | ND | 2.183 | 1.052 | 1.251 | 0.299 | 0.466 | ND | 1.478 | 0.549 | 2.080 |
| **IBD16** | 3.982 | 0.158 | 0.045 | 1.896 | 0.763 | 1.476 | 0.150 | 0.572 | 0.106 | 0.140 | 0.093 | 0.421 |
| **IBD17** | 0.828 | 0.216 | 0.013 | 0.103 | 0.500 | 0.935 | 0.089 | 0.472 | 0.376 | 0.023 | 0.866 | 1.767 |
| **IBD18** | 7.835 | 2.647 | 0.841 | 5.678 | 0.856 | 7.293 | 1.013 | 0.178 | 0.246 | 1.898 | 0.543 | 1.359 |
| **IBD19** | 7.650 | 4.023 | 3.384 | 5.601 | 4.403 | 2.074 | 0.812 | 0.246 | 1.112 | 0.292 | 1.669 | 0.527 |
| **IBD20** | 12.948 | 1.937 | ND | 2.250 | 0.354 | 2.604 | 0.102 | 1.023 | ND | 0.017 | 0.322 | 0.787 |
| **IBD21** | 1.310 | 1.261 | ND | 2.816 | 0.392 | 1.348 | 0.016 | 0.595 | ND | 0.068 | 0.240 | 0.220 |
| **IBD22** | 5.479 | 1.068 | ND | 165.430 | 1.342 | 0.460 | 0.025 | 7.555 | ND | 0.155 | 0.415 | 0.340 |
| **IBD23** | 3.922 | 0.908 | ND | 35.514 | 0.833 | 15.672 | 0.422 | 2.229 | ND | 0.255 | 1.279 | 2.055 |
| **IBD24** | 53.289 | 52.409 | ND | 47.468 | 4.403 | 31.664 | 0.575 | 5.490 | ND | 0.102 | 0.339 | 3.668 |
| **IBD25** | 5.969 | 1.604 | 0.308 | 4.963 | 1.410 | 3.033 | ND | ND | ND | ND | ND | ND |
| **IBD26** | 1.711 | 0.201 | 0.095 | 0.322 | 0.536 | 1.037 | 0.022 | 0.081 | 0.330 | 0.008 | 0.089 | 0.742 |
| **C1** | 0.926 | 0.256 | 0.127 | 1.316 | 0.848 | 1.389 | 1.437 | 0.852 | ND | 1.856 | 0.908 | 0.573 |
| **C2** | 1.390 | 1.997 | 0.640 | 0.368 | 0.878 | 0.834 | 1.185 | 2.323 | 0.218 | 1.756 | 3.403 | 1.933 |
| **C3** | 1.265 | 1.567 | 1.157 | 0.679 | 1.818 | 0.572 | 2.927 | 1.581 | 1.701 | 0.619 | 1.528 | 0.783 |
| **C4** | 1.181 | 0.284 | 0.441 | 1.097 | 1.343 | 0.791 | 0.031 | 0.089 | 0.309 | 0.398 | 0.466 | 0.048 |
| **C5** | 0.416 | 0.257 | 0.251 | 0.142 | 1.305 | 0.509 | 0.501 | 0.331 | 0.425 | 0.408 | 0.755 | 0.547 |
| **C6** | 0.386 | 1.606 | 0.286 | 0.592 | 1.279 | 0.743 | 0.026 | 0.758 | ND | 0.105 | 0.429 | 1.210 |
| **C7** | 1.050 | 0.526 | 2.667 | 0.415 | 1.202 | 0.352 | 0.021 | 0.273 | 0.049 | 0.067 | 0.505 | 1.108 |
| **C8** | 1.405 | 1.834 | 1.418 | 1.466 | 0.150 | 2.552 | 0.077 | 2.218 | 0.205 | 2.209 | 0.418 | 0.129 |
| **C9** | 0.981 | 0.673 | 2.014 | 2.924 | 0.177 | 1.258 | 2.796 | 0.576 | 4.093 | 1.582 | 0.589 | 2.669 |
